# Diseases of the Lungs

**Published:** 1886-04

**Authors:** 


DISEASES OF THE LUNGS (OF A SPECIFIC, NOT TUBERCULOUS NATURE), ACUTE BRONCHITIS, INFECTIOUS PNEUMONIA, GANGRENE, SYPHILIS, CANCER AND HYDATID OF THE LUNGS. By Germain Sée, member of the Academy of Medicine, member of the Faculty of Medicine, Physician to the Hotel Dieu, Paris, France. Translated by E. P. Hurd, M. D., member of the Massachusetts Medical Society, Vice-President of the Essex North Medical Society, one of the Physicians to the Anna Jaques Hospital, with Appendices by George M. Sternberg, M. D., Surgeon U. S. Army, and Professor Dujardin Beaumetz, member of the Academy of Medicine, Physician to the Hospital Cochin, Paris. New York. William Wood & Company, 56 and 58 Lafayette Place. 1885.

An elaborate preface of thirteen pages on Organisms, by Dr. Hurd, prepares the reader for that outgrowth of the germ theory of disease, which is presented in this volume, containing 305 pages, by Professor Germain Sée, on the Diseases of the Lungs, 15 pages, by Dr. George M. Sternberg, on the Pneumonia-Coccus of Friedlander, and 70 pages, by Dr. Dujardin Beaumetz, on Bacteria—all teeming with microbial philosophy.

